# Lower plasma cholesterol, LDL-cholesterol and LDL-lipoprotein subclasses in adult phenylketonuria (PKU) patients compared to healthy controls: results of NMR metabolomics investigation

**DOI:** 10.1186/s13023-020-1329-5

**Published:** 2020-02-27

**Authors:** Claire Cannet, Andrea Pilotto, Júlio César Rocha, Hartmut Schäfer, Manfred Spraul, Daniela Berg, Peter Nawroth, Christian Kasperk, Gwendolyn Gramer, Dorothea Haas, David Piel, Stefan Kölker, Georg Hoffmann, Peter Freisinger, Friedrich Trefz

**Affiliations:** 1 Bruker BioSpin GmbH, Rheinstetten, Germany; 2 Department of Neurodegeneration, Hertie Institute of Clinical Brain Research, University of Tübingen, Tübingen, Germany; 3 Neurology Unit, Department of Clinical and Experimental Sciences, University of Brescia, Brescia, Italy; 4 Parkinson’s Disease Rehabilitation Centre, FERB ONLUS S, Isidoro Hospital, Trescore Balneario, Italy; 5 Center for Health Technology and Services Research (CINTESIS), Porto, Portugal; 6 Nutrition & Metabolism, NOVA Medical School, Faculdade de Ciências Médicas, Universidade Nova de Lisboa, Lisbon, Portugal; 7 Department of Neurology, University-Hospital-Schleswig-Holstein, Christian-Albrechts-University, Kiel, Germany; 8 Department of Endocrinology and Metabolism, University Hospital, Heidelberg, Germany; 9 Department of Pediatrics, Centre for Pediatric and Adolescent Medicine, Division of Neuropaediatrics and Metabolic Medicine, University Hospital, Heidelberg, Germany; 10 Pediatrics, Reutlingen Hospital, Reutlingen, Germany; 11 Metabolic Consulting, Reutlingen, Germany

**Keywords:** Adult PKU, Treatment, Cholesterol, Lipoprotein subclasses, NMR

## Abstract

**Background:**

Phenylketonuria (PKU; OMIM#261600) is a rare metabolic disorder caused by mutations in the phenylalanine hydroxylase (PAH) gene resulting in high phenylalanine (Phe) in blood and brain. If not treated early this results in intellectual disability, behavioral and psychiatric problems, microcephaly, motor deficits, eczematous rash, autism, seizures, and developmental problems. There is a controversial discussion of whether patients with PKU have an additional risk for atherosclerosis due to interference of Phe with cholesterol synthesis and LDL-cholesterol regulation. Since cholesterol also plays a role in membrane structure and myelination, better insight into the clinical significance of the impact of Phe on lipoprotein metabolism is desirable. In 22 treated PKU patients (mean age 38.7 years) and 14 healthy controls (mean age 35.2 years), we investigated plasma with NMR spectroscopy and quantified 105 lipoprotein parameters (including lipoprotein subclasses) and 24 low molecular weight parameters. Analysis was performed on a 600 MHz Bruker AVANCE IVDr spectrometer as previously described.

**Results:**

Concurrent plasma Phe in PKU patients showed a wide range with a mean of 899 μmol/L (50–1318 μmol/L). Total cholesterol and LDL-cholesterol were significantly lower in PKU patients versus controls: 179.4 versus 200.9 mg/dL (*p* < 0.02) and 79.5 versus 104.1 mg/dL (*p* < 0.0038), respectively. PKU patients also had lower levels of 22 LDL subclasses with the greatest differences in LDL2 Apo-B, LDL2 Particle Number, LDL2-phospholipids, and LDL2-cholesterol (*p* < 0.0001). There was a slight negative correlation of total cholesterol and LDL-cholesterol with concurrent Phe level. VLDL5-free cholesterol, VLDL5-cholesterol, VLDL5-phospholipids, and VLDL4-free cholesterol showed a significant (*p* < 0.05) negative correlation with concurrent Phe level. There was no difference in HDL and their subclasses between PKU patients and controls. Tyrosine, glutamine, and creatinine were significantly lower in PKU patients compared to controls, while citric and glutamic acids were significantly higher.

**Conclusions:**

Using NMR spectroscopy, a unique lipoprotein profile in PKU patients can be demonstrated which mimics a non-atherogenic profile as seen in patients treated by statins.

## Background

Phenylketonuria (PKU; OMIM#261600) is a rare metabolic disorder caused by mutations in the phenylalanine hydroxylase (PAH) gene resulting in high phenylalanine (Phe) in blood and brain. If not treated early this results in intellectual disability, behavioral and psychiatric problems, microcephaly, motor deficits, eczematous rash, autism, seizures, and developmental problems. Although the PAH system converting Phe to tyrosine is well characterized, the pathophysiology of PKU and the impact of high Phe on the central nervous system is not well understood. Most theories focus on neurotransmitter depletion [1, 2], impaired brain protein synthesis [3, 4], and oxidative stress leading to early cell death and impaired mitochondrial function [5, 6]. A possible role of lipid metabolism was the focus of an investigation of essential fatty acids and a deficiency caused by the special PKU diet [7, 8]. A deficiency in cholesterol leading to hypomyelination as a possible cause of intellectual disability has been considered, but studies in patients are controversial and restricted to cholesterol measurements as recently summarized in a systematic review [9].

None of these studies have used modern NMR metabolomic lipoprotein profiling [10] as it is now widely used, e.g. in studies demonstrating statin effects in patients with cardiovascular disease [11]. We therefore applied this metabolomic technique in 22 adult classical PKU patients and 14 healthy age-matched controls. The aim of the study was to characterize the spectrum of cholesterol, LDL-cholesterol, HDL-cholesterol, lipoprotein subclasses, and low molecular weight parameters in comparison to controls to give further insight into the pathophysiology of brain damage in PKU [12]. In addition, the results should elucidate a possible cardiovascular risk in PKU patients [13] caused by an abnormal lipoprotein profile.

## Patients and methods

We performed plasma lipoprotein analysis to quantify 105 lipoprotein subclasses and 24 low molecular weight metabolites from the NMR spectra [10] in 22 treated adult PKU patients (16 females and 6 males) (Table 1), with a mean age of 38.7 (range 30–54) years, and a mean body mass index (BMI) of 27.2 (range 20.7–51.3) kg/m^2^. Controls (8 females and 6 males) had a mean age of 35.2 (range 30–45) years. Controls were also matched for social and educational level. BMI was slightly lower with a mean of 23.9 (range 21.3–29.8) kg/m^2^. Two out of 22 patients with triglycerides of 475 and 625 mg/dL respectively were excluded from lipoprotein evaluation. The high triglycerides were assumed not to be related to PKU or BMI (27.5 and 23.6 kg/m^2^, respectively). None of the patients or controls displayed clinical symptoms or medical histories indicating additional cardiovascular investigations. All patients were told to follow a Phe-restricted diet, but adherence to treatment recommendations was highly variable, yielding plasma Phe levels between 50 and 1318 μmol/L (mean 899). Plasma samples were drawn in the morning after an overnight fast and frozen at − 20 °C. Analysis was performed on a 600 MHz Bruker AVANCE IVDr spectrometer as previously described [10, 14]. Statistical analysis was performed using STATISTIKA.Ink. and SPSS 24.0.

**Table 1:** Patient characteristics

ID	Age	Gender	BMI	Actual Phe μmol/L	Allele 1	Allele 2	GPV^1^	Diet Adherence^2^
1	46	M	51.3	1318	p.R408W	IVS12 + 1G > A	0	0
2	35	F	21.2	1183	p.R261Q	IVS7 + 3 g > c	1.3	0
3	43	F	27	957	p.G239V	IVS10–11 g > a	n/a	1
4	45	M	26.8	1532	p.R158Q	p.R158Q	0	0
5	44	F	25	796	p.L48S	p.Y387H	2.4	1
6	30	F	21.8	402	n/a	n/a	n/a	1
7	45	M	23.3	730	p.R261Q	p.G272X	1.3	1
8	32	M	23.1	1017	p.S349P	p.L348V	n/a	1
9	39	F	31.2	648	p.L48S	p.R408W	2.4	1
10	42	F	21.3	51	p.P281L	IVS12 + 1G > A	0	1
11	54	F	29.6	238	p.L48S	IVS10–11 G > A	2.4	1
12	31	F	20.7	60	p.R158Q	p.R158Q	0	1
13	33	F	32	1013	IVS12 + 1G > A	IVS12 + 1G > A	0	0
14	38	F	41.8	1905	IVS10–11 g > a	p.R408W	0	0
15	41	F	21.2	142	p.R158W	p.R252Gfs*30	0	1
16	44	M	23.8	1409	p.P281L	p.R408W	0	1
17	31	F	24.9	929	p.R408W	IVS10-3C > T	0.9	1
18	45	F	22.4	349	p.R408W	p.R408W	0	1
19	36	F	31.2	1106	p.R158Q	p.R158Q	0	0
20	37	F	26.8	1207	p.F39L	p.R252W	1.4	0
21	30	M	27.5	866	p.R261Q	p.R408W	1.3	1
22	31	F	23.6	416	n/a	n/a	n/a	1
mean	**38.7**		**27.2**	**899**				

*BMI* body mass index, *GPV* genetic predicted value, *Phe* plasma phenylalanine at time of investigation.

^1^(www.biopku.org): < 5 predicts classical PKU

^2^Adherence to diet: 1, yes; 0, no.

Adherence to the diet was evaluated on basis of the diet prescription provided by the patient. 1: regular intake of low protein food, daily intake of amino acid Phe-free formula; 0: no intake of low protein food, irregular intake of amino acid formula

## Results

### Lipoprotein subclasses

**Fig. 1:**
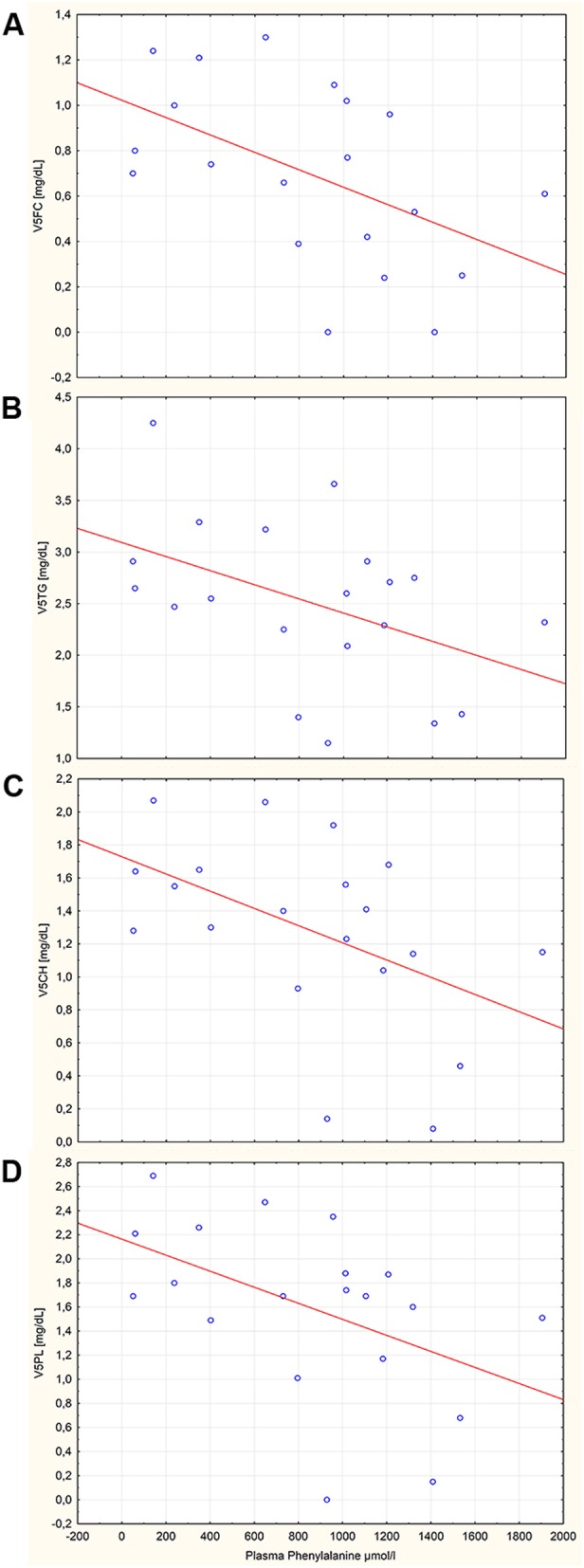
Correlation of plasma phenylalanine with (**a**) VLDL5-free cholesterol (R = -0.5.1, p < 0.02); (**b**) VLDL5-triglycerides (R = -0.45, *p* < 0.04); (**c**) VLDL5-cholesterol (R = -0.49, *p* < 0.02); and (**d**) VLDL5-phospholipids (R = -0.49, *p* < 0.04). Partial correlation to BMI is described in the text

There were significantly lower levels of total and LDL-cholesterol in PKU patients versus controls: 179.4 versus 200.9 mg/dL (*p* < 0.02) and 79.5 versus 104.1 mg/dL (*p* < 0.003), respectively. Total triglycerides differed widely among patients, but there was no difference between patients and controls (Complete list of results in Table S2). Significant disparities were restricted to LDL subclasses (Table 2) with the greatest differences in LDL2 Apo-B, LDL2 Particle Number, LDL2-phospholipids, and LDL2-cholesterol (*p* < 0.05). All other lipoprotein subclasses did not show significant differences between patients and controls (*p* > 0.05). There were no changes detectable in HDL and VLDL lipoproteins (Table S2). Adherence to diet did not correlate with lipoproteins but did with blood Phe levels: a significant negative correlation of plasma Phe with VLD5-cholesterol, VLDL5-free cholesterol, VLDL5-phospholipid, and VLDL5-triglyceride is shown in Fig. 1. Partial correlation of plasma Phe corrected for the effect of BMI with VLDL5-free cholesterol (R = -0.49.1, *p* = 0.024), VLDL5-triglycerides (R = -0.55, *p* = 0.009), VLDL5-cholesterol (R = -0.54, *p* = 0.012), and VLDL5- phospholipids (R = -0.58, *p* = 0.006) confirmed the negative impact of Phe on these VLDL subclasses. Cholesterol and LDL-cholesterol also showed a negative correlation to Phe; however, this was not statistically significant.

**Table 2:** Significant differences of plasma lipoproteins in PKU patients and controls (t-test). Explanation of terminology is presented in Table S1

Lipoprotein	Controls (*n* = 14)		Patients (*n* = 20)		*p*-value
	Mean	SD	Mean	SD	
L2AB [mg/dL]	9.6	2.8	5.2	2.9	0.00005
L2PN [nmol/L]	173.9	51.6	93.9	53.5	0.00005
L2PL [mg/dL]	9.7	2.6	5.4	3.1	0.00007
L2CH [mg/dL]	17	5.5	8.3	6.4	0.00009
L2FC [mg/dL]	5.4	1.6	3.1	2	0.00053
L1CH [mg/dL]	24.8	3.8	18.2	6.4	0.00078
L1AB [mg/dL]	13.2	2	10.2	2.9	0.00105
L1PN [nmol/L]	239.1	36.6	185.1	53.2	0.00106
L1PL [mg/dL]	14.2	2	11.2	3	0.00115
L1FC [mg/dL]	7.4	1.3	5.6	2	0.00239
LDPL [mg/dL]	60.9	14.2	48.9	10.9	0.00368
LDCH [mg/dL]	104.1	30	79.5	21.8	0.00382
LDFC [mg/dL]	31	7.8	24.1	6.9	0.00414
L3CH [mg/dL]	14.5	7	8.6	5.6	0.0045
L3AB [mg/dL]	8.7	3.9	5.5	3.1	0.00554
L3PN [nmol/L]	158	71.6	100.6	56.1	0.00555
L3PL [mg/dL]	8.5	3.5	5.5	3	0.00579
LDPN [nmol/L]	1229.8	335.9	994.6	224	0.00815
LDAB [mg/dL]	67.6	18.5	54.7	12.3	0.00815
L3TG [mg/dL]	2.5	0.7	2	0.7	0.01909
L3FC [mg/dL]	4.6	1.7	3.3	1.8	0.0219
TPCH [mg/dL]	201	33.2	179.4	28.1	0.02204

### Low molecular weight metabolites

Low molecular weight metabolites besides Phe revealed significant differences for the following metabolites (PKU versus controls, Table 3): glutamine (611 versus 690 μmol/L, *p* < 0.01); creatinine (74.8 versus 86.1 μmol/L, p < 0.01); and tyrosine (42.5 versus 56.9 μmol/L, *p* < 0.005). Glutamic acid as well as citric acid were significantly higher in PKU patients versus controls: 87.6 versus 50.5 μmol/L (*p* < 0.015) and 186.9 versus 157.2 μmol/L (*p* < 0.019), respectively.

**Table 3:** Results of low molecular plasma parameters in adult PKU patients compared to controls. Significant differences were set to *p* < 0.05 (t-test)

Metabolite (μmol/L)	Controls (*n* = 14)		Patients (*n* = 22)		*p*-value
	Mean	SD	Mean	SD	
Phenylalanine	49.2	10.1	830.7	503	0.00000
DL-Tyrosine	56.9	7.8	42.6	18.7	0.00514
Glutamine	690.6	88.4	611.4	99.1	0.01013
Glutamic acid	50.5	29.2	87.7	57.3	0.01596
Creatinine	86.1	12.7	74.8	16	0.01647
Citric acid	157.2	30.9	186.9	45.4	0.01952
3-Hydroxybutyric acid	47.6	34	83.9	102.3	0.10514
Acetoacetic acid	10.1	14.3	19.8	30	0.13468
L-Isoleucine	50.1	15.6	45.3	11.5	0.14610
Creatine	15.6	10.2	11.8	11.2	0.15723
Histidine	76.9	25.5	90.7	72	0.24784
Ethanol	107.2	55.5	117.1	45.1	0.28115
Pyruvic acid	89	38.5	96.7	38.9	0.28319
Alanine	415.5	116.5	432.8	89.7	0.31004
Trimethylamine-N-oxide	21.3	16.9	18.3	17.7	0.31144
Leucine	93.1	23.5	89.1	24.4	0.31443
Formic acid	17.5	6	16.7	4.5	0.32376
Threonine	59.8	99.2	69.5	64.2	0.36175
Acetone	23.3	10.5	21.5	18.7	0.37320
Valine	218.4	43.9	222.4	60.4	0.41777
Glycine	311.5	101.2	318.7	99.8	0.41813
Acetic acid	19.1	16	17.9	17.6	0.41959
D-Glucose	4904.1	481	4830.8	1188	0.41409
Lactic acid	2409.7	405.2	3033	1646.8	0.08787

**Fig. 2:**
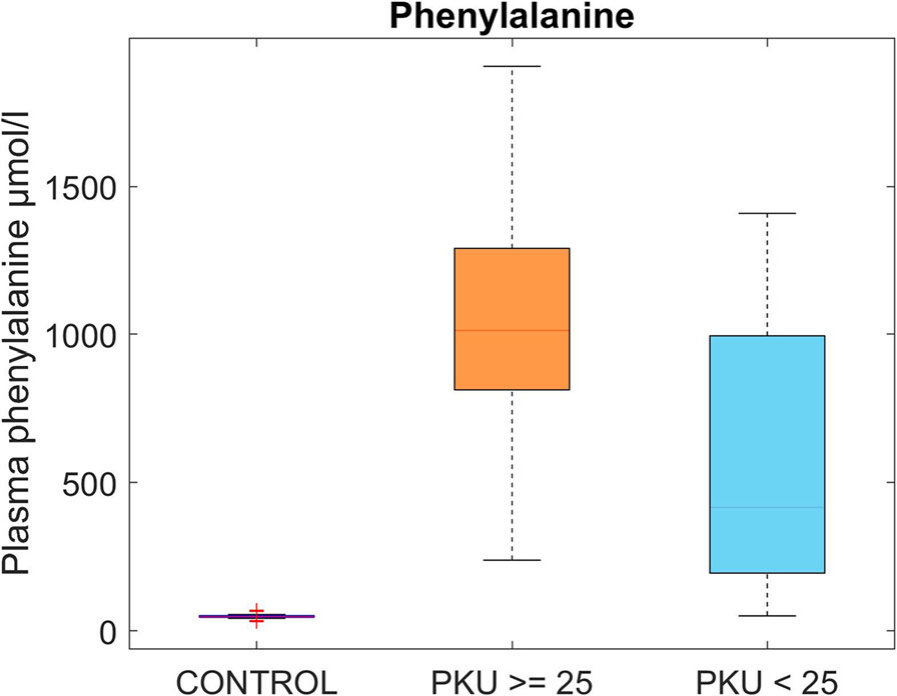
Phenylalanine control at time of investigation grouped by body mass index (BMI) ≥25 (*n* = 10) and < 25 (*n* = 12) in PKU patients versus controls (*n* = 14)

BMI did not show a significant influence on the lipoprotein subclasses in the patients investigated except quality of dietary treatment; patients with higher BMI had less optimal Phe control than those with a lower BMI (Fig. 2).

## Discussion

The data show an impact of Phe on lipoprotein concentration in plasma indicating a negative influence on cholesterol synthesis or LDL regulation. Animal models have demonstrated decreases in 3-hydroxy-3-methylglutaryl-CoA reductase (HMGR) and mevalonate-5-pyrophosphate decarboxylase in liver and brain by high Phe [15] leading to impaired cholesterol synthesis. It was speculated that this impairment may lead to hypomyelination and mental retardation in untreated PKU patients. Later on, it was shown that HMGR activity was not impaired in the liver in a PAH (enu2) genetic mouse model, but a reduction of HMGR activity by 40% was found in oligodendrocytes of the forebrain in the hypomyelinated tracts [16]. In summary, there have been inconsistent findings about the impact of Phe on cholesterol concentration: 12 studies demonstrated lower levels of cholesterol, while 6 studies did not, as recently published in a systematic review by Montoya Parra et al. [9].

There is a unique lipoprotein profile pattern in our PKU patients compared to controls. Significant disparities are limited to cholesterol, LDL-cholesterol, and LDL subclasses. Interpretation of the intergroup difference is difficult since there is only a small negative correlation of Phe on total cholesterol and LDL-cholesterol, which is not statistically significant (data not shown). In contrast, there is a significant negative correlation between plasma Phe and higher densities of VLDL subfractions (VLDL5 corresponding to 1.006 kg/L) as shown in Fig. 1 A–D also when corrected by BMI. Low cholesterol and LDL-cholesterol have also been observed by Couce et al. [17] when comparing patients with hyperphenylalaninemia and classical PKU. Since well-treated PKU patients are treated by a vegan-like diet supplemented with an artificial Phe-free amino acid mixture, dietary effects on the lipid pattern cannot be excluded [9]. While HDL-cholesterol can be lowered by a vegan diet, LDL-cholesterol levels are not influenced by a vegan diet [18]. However, in a meta-analysis [19], it is evident that cholesterol, LDL-cholesterol and HDL-cholesterol are reduced by a vegan diet. Our patients with a less well-controlled diet leading to higher Phe levels should have higher intakes of natural protein and therefore higher risks of elevated lipoproteins. As shown by the extensive lipoprotein subclass analysis, they had lower total cholesterol and LDL-cholesterol but not HDL-cholesterol. This leads to the discussion of how cholesterol and LDL-cholesterol may be influenced/decreased in PKU patients.

Regulation of LDL-cholesterol is a complex mechanism as demonstrated in a comprehensive review by Goldstein and Brown [20]. Cells obtain cholesterol from endogenous synthesis via HMG CoA, receptor-mediated uptake, and lysosomal hydrolysis of LDL-cholesterol. Statins reduce cholesterol synthesis by inhibition of HMG-reductase activity. High Phe may mimic this statin effect. Regulation of the LDL receptor gene is mediated by sterol regulatory element-binding protein-1 (SREBP) transcription factors. When entering the nucleus, SREBPs also activate endogenous cholesterol biosynthesis. A low cholesterol diet as present in PKU patients may lead to activation of SREBPs which activate LDL receptor transcription and HMGR activity, thereby increasing cholesterol synthesis and decreasing LDL [20]. Another player of LDL regulation is proprotein convertase subtilisin/kexin type 9 (PCSK9) [21, 22]. Mutations in the gene of this protein, which interferes with the LDL receptor, may destroy the LDL receptor or partially inactivate PCSK9, thus reducing plasma LDL levels. No data are available demonstrating whether high Phe interferes with this protein function. In summary, the LDL lowering mechanism in PKU patients is unclear and needs further investigation [9]. The statin-like effect of high Phe may also be the cause of higher concentrations of citrate and glutamic acid in patients compared to controls, as inhibition of HMGR leads to an increase of HMG-CoA which feeds acetyl CoA into the citric cycle.

The role of the vegan diet on lipoprotein profile in our PKU patients cannot be totally excluded. However, a negative impact of Phe on cholesterol, LDL-cholesterol, VLDL-cholesterol (except HDL-cholesterol) and their subclasses indicate a key role of elevated Phe on the lipoprotein profile with great similarities to that seen in patients with high cholesterol using statins [11].

Low molecular mass metabolites showed that in the patients investigated there was no evidence of catabolism as indicated (e.g. by elevated ketones). However, acetoacetic and 3-OH-butyric acid tended to be somewhat higher but reached no significant differences (Table 3). The same was true for lactic acid, which showed (due to measuring plasma) higher levels for both controls and patients than found in capillary blood. Glutamine was significantly lower in PKU patients as previously described [23] and may be due to increased excretion of N-acetylglutamine in urine (due to high phenylacetic acid and glutamine excretion in urine). Clinical significance of this difference is unclear. In earlier studies this was discussed as another possible cause of mental retardation in PKU siblings and may be important for the developing brain in PKU infants (“glutamine depletion hypothesis” [24]). Although quality of treatment in our patients was highly variable, most of them used amino acid supplements, which are devoid of Phe but enriched in tyrosine and other essential amino acids. Despite this supplementation, tyrosine was significantly lower in PKU patients which may contribute to neurotransmitter depletion and impaired brain protein synthesis [1, 2, 25, 26]. No significant differences were found for branched chain amino acids. This may be due to the number of relatively well-treated patients in our sample (11 of 22 had Phe levels < 900 μmol/L, a value below the recommended target Phe level for German adult PKU patients [27]. Furthermore, six patients had Phe levels < 600 μmol/L, a target value recommended in the European guidelines for PKU [28, 29]).

Chronic kidney disease (CKD) was recently discussed as a possible comorbidity of patients with classical PKU [30, 31]. In our patients, there was a significant difference in creatinine. The low creatinine levels may be due to lower muscle mass [32]. Although renal functional tests were not performed, it indicates that at least in our patients CKD was not (yet) present.

### Strengths and limitations

Our study was limited by the availability of patients’ dietary records over 3 days. Therefore, correlations to natural protein intake could not be made and the additional impact of a vegan-like diet could not be excluded.

However, our results demonstrate for the first time the power of NMR metabolomic investigation in PKU patients in contrast to the conventional time-consuming lipoprotein analysis as shown recently [33]. Besides imbalances in the lipoprotein pattern revealing lower levels of cholesterol and LDL subclasses, together with low molecular weight analysis, this method allows a more comprehensive monitoring of PKU patients as also demonstrated in another (urinary) metabolomics approach recently [34]. Our findings of low LDL-cholesterol and low LDL subclass levels in these patients suggest this may protect them from early atherosclerosis and the lipoprotein profile is non-atherogenic. Since cardiovascular comorbidities have been described by our group and others [33, 35], it cannot be excluded that patients may suffer from other risk factors such as type 2 diabetes, obesity, metabolic syndrome, or increased oxidative stress caused by high Phe or the Phe-restricted low protein diet. In addition, a possible direct effect of Phe on the arterial wall leading to arterial stiffness was shown recently [13, 35]. The mechanism of decreased cholesterol and LDL lipoprotein subclasses by increased Phe needs further investigation.

## Conclusions

Using NMR spectroscopy, a unique lipoprotein profile in PKU patients can be demonstrated which mimics a non-atherogenic profile as seen in patients treated by statins.

## Supplementary information

**Additional file 1: Table S1.** Terminology of 117 lipoprotein parameters measured with the lipoprotein distribution (LPD) prediction method. The 105 measured parameters are presented in the first table, while the 12 parameters, which are calculated from the original ones, are presented at the end of the table (From https://pubs.acs.org/doi/abs/10.1021/acs.analchem.8b02412 with changes). **Table S2**. Results of low molecular weight metabolites in PKU patients and controls: mean, standard deviation and p significance (t-test) are presented in the first table; lipoprotein subclasses (explanation in Table S1) are presented in the second table. **Figure S1.** Correlation between 2 years mean blood phenylalanine (Phe) level and concurrent Phe level in 7 PKU patients (historical blood Phe levels measured in dried blood by tandem mass spectrometry).

## Data Availability

All data are available in the supplementary material.
