# Supplementary material for: Lower plasma cholesterol, LDL-cholesterol and LDL-lipoprotein subclasses in adult phenylketonuria (PKU) patients compared to healthy controls: results of NMR metabolomics investigation
Source: Orphanet J Rare Dis. 2020 Feb 27;15:61. doi: 10.1186/s13023-020-1329-5 (PMC7047385; doi:10.1186/s13023-020-1329-5)
Supplement: Supplementary file 1 — Additional file 1: Table S1. Terminology of 117 lipoprotein parameters measured with the lipoprotein distribution (LPD) prediction method. The 105 measured parameters are presented in the first table, while the 12 parameters, which are calculated from the original ones, are presented at the end of the table (From https://pubs.acs.org/doi/abs/10.1021/acs.analchem.8b02412 with changes). Table S2. Results of low molecular weight metabolites in PKU patients and controls: mean, standard deviation and p significance (t-test) are presented in the first table; lipoprotein subclasses (explanation in Table S1) are presented in the second table. Figure S1. Correlation between 2 years mean blood phenylalanine (Phe) level and concurrent Phe level in 7 PKU patients (historical blood Phe levels measured in dried blood by tandem mass spectrometry). [file 13023_2020_1329_MOESM1_ESM.docx]

**Table S1.** Terminology of 117 lipoprotein parameters measured with the lipoprotein distribution (LPD) prediction method. The 105 measured parameters are presented in the first table, while the 12 parameters, which are calculated from the original ones, are presented at the end of the table (From <https://pubs.acs.org/doi/abs/10.1021/acs.analchem.8b02412> with changes).

| Matrix | Measured Analytes | Name |
| --- | --- | --- |
| Total Plasma | Triglycerides | TPTG |
| Total Plasma | Cholesterol | TPCH |
| Total Plasma | Free Cholesterol | TPFC |
| Total Plasma | Apo-A1 | TPA1 |
| Total Plasma | Apo-A2 | TPA2 |
| Total Plasma | Apo-B | TPAB |
| VLDL | Triglycerides | VLTG |
| VLDL | Cholesterol | VLCH |
| VLDL | Free Cholesterol | VLFC |
| VLDL | Phospholipids | VLPL |
| VLDL | Apo-B | VLAB |
| IDL | Triglycerides | IDTG |
| IDL | Cholesterol | IDCH |
| IDL | Free Cholesterol | IDFC |
| IDL | Phospholipids | IDPL |
| IDL | Apo-B | IDAB |
| LDL | Triglycerides | LDTG |
| LDL | Cholesterol | LDCH |
| LDL | Free Cholesterol | LDFC |
| LDL | Phospholipids | LDPL |
| LDL | Apo-B | LDAB |
| HDL | Triglycerides | HDTG |
| HDL | Cholesterol | HDCH |
| HDL | Free Cholesterol | HDFC |
| HDL | Phospholipids | HDPL |
| HDL | Apo-A1 | HDA1 |
| HDL | Apo-A2 | HDA2 |
| VLDL-1 | Triglycerides | V1TG |
| VLDL-1 | Cholesterol | V1CH |
| VLDL-1 | Free Cholesterol | V1FC |
| VLDL-1 | Phospholipids | V1PL |
| VLDL-2 | Triglycerides | V2TG |
| VLDL-2 | Cholesterol | V2CH |
| VLDL-2 | Free Cholesterol | V2FC |
| VLDL-2 | Phospholipids | V2PL |
| VLDL-3 | Triglycerides | V3TG |
| VLDL-3 | Cholesterol | V3CH |
| VLDL-3 | Free Cholesterol | V3FC |
| VLDL-3 | Phospholipids | V3PL |
| VLDL-4 | Triglycerides | V4TG |
| VLDL-4 | Cholesterol | V4CH |
| VLDL-4 | Free Cholesterol | V4FC |
| VLDL-4 | Phospholipids | V4PL |
| VLDL-5 | Triglycerides | V5TG |
| VLDL-5 | Cholesterol | V5CH |
| VLDL-5 | Free Cholesterol | V5FC |
| VLDL-5 | Phospholipids | V5PL |
| VLDL-6 | Triglycerides | V6TG |
| VLDL-6 | Cholesterol | V6CH |
| VLDL-6 | Free Cholesterol | V6FC |
| VLDL-6 | Phospholipids | V6PL |
| LDL-1 | Triglycerides | L1TG |
| LDL-1 | Cholesterol | L1CH |
| LDL-1 | Free Cholesterol | L1FC |
| LDL-1 | Phospholipids | L1PL |
| LDL-1 | Apo-B | L1AB |
| LDL-2 | Triglycerides | L2TG |
| LDL-2 | Cholesterol | L2CH |
| LDL-2 | Free Cholesterol | L2FC |
| LDL-2 | Phospholipids | L2PL |
| LDL-2 | Apo-B | L2AB |
| LDL-3 | Triglycerides | L3TG |
| LDL-3 | Cholesterol | L3CH |
| LDL-3 | Free Cholesterol | L3FC |
| LDL-3 | Phospholipids | L3PL |
| LDL-3 | Apo-B | L3AB |
| LDL-4 | Triglycerides | L4TG |
| LDL-4 | Cholesterol | L4CH |
| LDL-4 | Free Cholesterol | L4FC |
| LDL-4 | Phospholipids | L4PL |
| LDL-4 | Apo-B | L4AB |
| LDL-5 | Triglycerides | L5TG |
| LDL-5 | Cholesterol | L5CH |
| LDL-5 | Free Cholesterol | L5FC |
| LDL-5 | Phospholipids | L5PL |
| LDL-5 | Apo-B | L5AB |
| LDL-6 | Triglycerides | L6TG |
| LDL-6 | Cholesterol | L6CH |
| LDL-6 | Free Cholesterol | L6FC |
| LDL-6 | Phospholipids | L6PL |
| LDL-6 | Apo-B | L6AB |
| HDL-1 | Triglycerides | H1TG |
| VLDL-3 | Phospholipids | V6PL |
| VLDL-4 | Triglycerides | L1TG |
| VLDL-4 | Cholesterol | L1CH |
| VLDL-4 | Free Cholesterol | L1FC |
| VLDL-4 | Phospholipids | L1PL |
| VLDL-5 | Apo-B | L1AB |
| VLDL-5 | Triglycerides | L2TG |
| VLDL-5 | Cholesterol | L2CH |
| VLDL-5 | Free Cholesterol | L2FC |
| VLDL-6 | Phospholipids | L2PL |
| VLDL-6 | Apo-B | L2AB |
| VLDL-6 | Triglycerides | L3TG |
| VLDL-6 | Cholesterol | L3CH |
| LDL-1 | Free Cholesterol | L3FC |
| LDL-1 | Phospholipids | L3PL |
| LDL-1 | Apo-B | L3AB |
| LDL-1 | Triglycerides | L4TG |
| LDL-1 | Cholesterol | L4CH |
| LDL-2 | Free Cholesterol | L4FC |
| LDL-2 | Cholesterol | L2CH |
| LDL-2 | Free Cholesterol | L2FC |
| LDL-2 | Phospholipids | L2PL |
| LDL-2 | Apo-B | L2AB |
| LDL-3 | Triglycerides | L3TG |
| LDL-3 | Cholesterol | L3CH |
| LDL-3 | Free Cholesterol | L3FC |
| LDL-3 | Phospholipids | L3PL |
| LDL-3 | Apo-B | L3AB |
| LDL-4 | Triglycerides | L4TG |
| LDL-4 | Cholesterol | L4CH |
| LDL-4 | Free Cholesterol | L4FC |
| LDL-4 | Phospholipids | L4PL |
| LDL-4 | Apo-B | L4AB |
| LDL-5 | Triglycerides | L5TG |
| LDL-5 | Cholesterol | L5CH |
| LDL-5 | Free Cholesterol | L5FC |
| LDL-5 | Phospholipids | L5PL |
| LDL-5 | Apo-B | L5AB |
| LDL-6 | Triglycerides | L6TG |
| LDL-6 | Cholesterol | L6CH |
| LDL-6 | Free Cholesterol | L6FC |
| LDL-6 | Phospholipids | L6PL |
| LDL-6 | Apo-B | L6AB |
| HDL-1 | Triglycerides | H1TG |
| HDL-1 | Cholesterol | H1CH |
| HDL-1 | Free Cholesterol | H1FC |
| HDL-1 | Phospholipids | H1PL |
| HDL-1 | Apo-A1 | H1A1 |
| HDL-1 | Apo-A2 | H1A2 |
| HDL-2 | Triglycerides | H2TG |
| HDL-2 | Cholesterol | H2CH |
| HDL-2 | Free Cholesterol | H2FC |
| HDL-2 | Phospholipids | H2PL |
| HDL-2 | Apo-A1 | H2A1 |
| HDL-2 | Apo-A2 | H2A2 |
| HDL-3 | Triglycerides | H3TG |
| HDL-3 | Cholesterol | H3CH |
| HDL-3 | Free Cholesterol | H3FC |
| HDL-3 | Phospholipids | H3PL |
| HDL-3 | Apo-A1 | H3A1 |
| HDL-3 | Apo-A2 | H3A2 |
| HDL-4 | Triglycerides | H4TG |
| HDL-4 | Cholesterol | H4CH |
| HDL-4 | Free Cholesterol | H4FC |
| HDL-4 | Phospholipids | H4PL |
| HDL-4 | Apo-A1 | H4A1 |
| HDL-3 | Apo-A2 | H3A2 |
| HDL-4 | Triglycerides | H4TG |
| HDL-4 | Cholesterol | H4CH |
| HDL-4 | Free Cholesterol | H4FC |
| HDL-4 | Phospholipids | H4PL |
| HDL-4 | Apo-A1 | H4A1 |
| HDL-4 | Apo-A2 | H4A2 |

| LDL/HDL | LDL-Chol/HDL-Chol | LDHD |
| --- | --- | --- |
| Apo-B/Apo-A1 | Apo-B/Apo-A1 | ABA1 |
| Total Plasma | Particle Number | TBPN |
| VLDL | Particle Number | VLPN |
| IDL | Particle Number | IDPN |
| LDL | Particle Number | LDPN |
| LDL-1 | Particle Number | L1PN |
| LDL-2 | Particle Number | L2PN |
| LDL-3 | Particle Number | L3PN |
| LDL-4 | Particle Number | L4PN |
| LDL-5 | Particle Number | L5PN |
| LDL-6 | Particle Number | L6PN |

| Table S2. Results of low molecular weight metabolites in PKU patients and controls: mean, standard deviation and p significance (t-test) are presented in the first table; lipoprotein subclasses (explanation in Table S1) are presented in the second table. | | | | | |
| --- | --- | --- | --- | --- | --- |
| Metabolite (µmol/L) | **Controls** | | **Patients** | |  |
|  | **Mean** | **SD** | **Mean** | **SD** | **p-value** |
| 3-Hydroxybutyric acid | 47.6 | 34.0 | 83.9 | 102.3 | 0.10514 |
| ABA1 [-/-] no unit | 0.5 | 0.2 | 0.5 | 0.2 | 0.10137 |
| Acetic acid | 19.1 | 16.0 | 17.9 | 17.6 | 0.41959 |
| Acetoacetic acid | 10.1 | 14.3 | 19.8 | 30.0 | 0.13468 |
| Acetone | 23.3 | 10.5 | 21.5 | 18.7 | 0.37320 |
| Alanine | 415.5 | 116.5 | 432.8 | 89.7 | 0.31004 |
| Citric acid | 157.2 | 30.9 | 186.9 | 45.4 | 0.01952 |
| Creatine | 15.6 | 10.2 | 11.8 | 11.2 | 0.15723 |
| Creatinine | 86.1 | 12.7 | 74.8 | 16.0 | 0.01647 |
| D-Glucose | 4904.1 | 481.0 | 4830.8 | 1188.0 | 0.41409 |
| DL-Tyrosine | 56.9 | 7.8 | 42.6 | 18.7 | 0.00514 |
| Ethanol | 107.2 | 55.5 | 117.1 | 45.1 | 0.28115 |
| Formic acid | 17.5 | 6.0 | 16.7 | 4.5 | 0.32376 |
| Histidine | 76.9 | 25.5 | 90.7 | 72.0 | 0.24784 |
| Glutamic acid | 50.5 | 29.2 | 87.7 | 57.3 | 0.01596 |
| Glutamine | 690.6 | 88.4 | 611.4 | 99.1 | 0.01013 |
| Glycine | 311.5 | 101.2 | 318.7 | 99.8 | 0.41813 |
| Lactic acid | 2409.7 | 405.2 | 3033.0 | 1646.8 | 0.08787 |
| Leucine | 93.1 | 23.5 | 89.1 | 24.4 | 0.31443 |
| L-Isoleucine | 50.1 | 15.6 | 45.3 | 11.5 | 0.14610 |
| Phenylalanine | 49.2 | 10.1 | 830.7 | 503.0 | 0.00000 |
| Pyruvic acid | 89.0 | 38.5 | 96.7 | 38.9 | 0.28319 |
| Threonine | 59.8 | 99.2 | 69.5 | 64.2 | 0.36175 |
| Trimethylamine-N-oxide | 21.3 | 16.9 | 18.3 | 17.7 | 0.31144 |
| Valine | 218.4 | 43.9 | 222.4 | 60.4 | 0.41777 |
|  | | | | | |
| H1A1 [mg/dL] | 29.8 | 21.0 | 27.2 | 25.3 | 0.37771 |
| H1A2 [mg/dL] | 3.4 | 2.0 | 3.3 | 3.4 | 0.45988 |
| H1CH [mg/dL] | 17.8 | 11.6 | 16.6 | 13.5 | 0.39485 |
| H1FC [mg/dL] | 4.8 | 2.7 | 4.4 | 3.1 | 0.35901 |
| H1PL [mg/dL] | 23.6 | 14.7 | 22.3 | 17.7 | 0.41244 |
| H1TG [mg/dL] | 3.4 | 2.4 | 4.5 | 5.8 | 0.25740 |
| H2A1 [mg/dL] | 21.5 | 6.5 | 22.2 | 9.6 | 0.41184 |
| H2A2 [mg/dL] | 4.3 | 1.3 | 4.3 | 1.8 | 0.48100 |
| H2CH [mg/dL] | 9.0 | 3.2 | 8.4 | 3.0 | 0.29961 |
| H2FC [mg/dL] | 2.3 | 0.8 | 2.2 | 0.7 | 0.31092 |
| H2PL [mg/dL] | 14.8 | 5.1 | 14.2 | 5.2 | 0.37311 |
| H2TG [mg/dL] | 1.8 | 0.8 | 2.3 | 2.1 | 0.21523 |
| H3A1 [mg/dL] | 29.4 | 6.0 | 30.3 | 7.7 | 0.35092 |
| H3A2 [mg/dL] | 7.4 | 1.5 | 7.7 | 1.8 | 0.30587 |
| H3CH [mg/dL] | 11.3 | 2.3 | 11.2 | 3.0 | 0.49307 |
| H3FC [mg/dL] | 2.6 | 0.8 | 2.5 | 0.8 | 0.28296 |
| H3PL [mg/dL] | 19.1 | 3.9 | 19.0 | 4.9 | 0.47198 |
| H3TG [mg/dL] | 2.3 | 0.6 | 2.8 | 1.8 | 0.13181 |
| H4A1 [mg/dL] | 79.1 | 9.5 | 81.0 | 12.6 | 0.31526 |
| H4A2 [mg/dL] | 21.4 | 3.6 | 22.0 | 3.2 | 0.32385 |
| H4CH [mg/dL] | 21.4 | 3.1 | 21.0 | 5.1 | 0.39124 |
| H4FC [mg/dL] | 4.2 | 1.0 | 4.1 | 1.3 | 0.44038 |
| H4PL [mg/dL] | 31.1 | 3.6 | 30.9 | 6.1 | 0.45947 |
| H4TG [mg/dL] | 3.7 | 0.8 | 4.5 | 2.0 | 0.07771 |
| HDA1 [mg/dL] | 159.5 | 34.5 | 159.5 | 38.8 | 0.49992 |
| HDA2 [mg/dL] | 36.8 | 4.9 | 37.6 | 6.8 | 0.35953 |
| HDCH [mg/dL] | 61.1 | 16.5 | 58.0 | 16.3 | 0.28742 |
| HDCH [mg/dL] | 61.1 | 16.5 | 58.0 | 16.3 | 0.28742 |
| HDFC [mg/dL] | 13.0 | 5.1 | 12.2 | 4.3 | 0.31794 |
| HDPL [mg/dL] | 88.9 | 23.0 | 87.0 | 23.5 | 0.40545 |
| HDTG [mg/dL] | 11.0 | 3.8 | 13.9 | 11.3 | 0.18006 |
| IDAB [mg/dL] | 5.0 | 2.2 | 5.5 | 3.4 | 0.32242 |
| IDCH [mg/dL] | 11.7 | 6.8 | 13.4 | 11.1 | 0.29991 |
| IDFC [mg/dL] | 3.5 | 1.9 | 4.0 | 3.1 | 0.29453 |
| IDPL [mg/dL] | 6.5 | 2.9 | 8.0 | 6.4 | 0.20541 |
| IDPN [nmol/L] | 91.2 | 40.0 | 99.8 | 61.4 | 0.32189 |
| IDTG [mg/dL] | 8.1 | 5.5 | 16.4 | 24.8 | 0.11286 |
| L1AB [mg/dL] | 13.2 | 2.0 | 10.2 | 2.9 | 0.00105 |
| L1CH [mg/dL] | 24.8 | 3.8 | 18.2 | 6.4 | 0.00078 |
| L1FC [mg/dL] | 7.4 | 1.3 | 5.6 | 2.0 | 0.00239 |
| L1PL [mg/dL] | 14.2 | 2.0 | 11.2 | 3.0 | 0.00115 |
| L1PN [nmol/L] | 239.1 | 36.6 | 185.1 | 53.2 | 0.00106 |
| L1TG [mg/dL] | 5.4 | 1.5 | 5.9 | 3.0 | 0.27381 |
| L2AB [mg/dL] | 9.6 | 2.8 | 5.2 | 2.9 | 0.00005 |
| L2CH [mg/dL] | 17.0 | 5.5 | 8.3 | 6.4 | 0.00009 |
| L2FC [mg/dL] | 5.4 | 1.6 | 3.1 | 2.0 | 0.00053 |
| L2PL [mg/dL] | 9.7 | 2.6 | 5.4 | 3.1 | 0.00007 |
| L2PN [nmol/L] | 173.9 | 51.6 | 93.9 | 53.5 | 0.00005 |
| L2TG [mg/dL] | 2.3 | 0.5 | 2.0 | 0.6 | 0.04433 |
| L3AB [mg/dL] | 8.7 | 3.9 | 5.5 | 3.1 | 0.00554 |
| L3CH [mg/dL] | 14.5 | 7.0 | 8.6 | 5.6 | 0.00450 |
| L3FC [mg/dL] | 4.6 | 1.7 | 3.3 | 1.8 | 0.02190 |
| L3PL [mg/dL] | 8.5 | 3.5 | 5.5 | 3.0 | 0.00579 |
| L3PN [nmol/L] | 158.0 | 71.6 | 100.6 | 56.1 | 0.00555 |
| L3TG [mg/dL] | 2.5 | 0.7 | 2.0 | 0.7 | 0.01909 |
| L4AB [mg/dL] | 9.0 | 5.6 | 7.6 | 4.7 | 0.20723 |
| L4CH [mg/dL] | 13.4 | 8.6 | 11.0 | 7.2 | 0.18527 |
| L4FC [mg/dL] | 3.9 | 2.1 | 3.4 | 1.8 | 0.24289 |
| L4PL [mg/dL] | 7.8 | 4.5 | 6.7 | 3.9 | 0.21582 |
| L4PN [nmol/L] | 163.8 | 102.4 | 137.6 | 86.2 | 0.20739 |
| L4TG [mg/dL] | 2.3 | 1.1 | 2.2 | 1.0 | 0.38255 |
| L5AB [mg/dL] | 11.1 | 5.7 | 10.2 | 3.9 | 0.30163 |
| L5CH [mg/dL] | 15.1 | 8.4 | 13.5 | 5.8 | 0.25267 |
| L5FC [mg/dL] | 4.3 | 2.0 | 4.0 | 1.5 | 0.28439 |
| L5PL [mg/dL] | 8.5 | 4.2 | 7.8 | 2.9 | 0.27426 |
| L5PN [nmol/L] | 201.3 | 104.0 | 186.1 | 70.0 | 0.30140 |
| L5TG [mg/dL] | 2.5 | 1.0 | 2.7 | 1.0 | 0.33310 |
| L6AB [mg/dL] | 17.3 | 5.3 | 18.3 | 8.9 | 0.35995 |
| L6CH [mg/dL] | 20.9 | 6.3 | 21.1 | 8.7 | 0.47459 |
| L6FC [mg/dL] | 5.3 | 1.3 | 5.3 | 1.9 | 0.44336 |
| L6PL [mg/dL] | 12.2 | 3.0 | 12.5 | 4.7 | 0.41653 |
| L6PN [nmol/L] | 314.6 | 96.9 | 332.1 | 162.3 | 0.35959 |
| L6TG [mg/dL] | 3.8 | 1.0 | 4.4 | 3.0 | 0.22664 |
| LDAB [mg/dL] | 67.6 | 18.5 | 54.7 | 12.3 | 0.00815 |
| LDCH [mg/dL] | 104.1 | 30.0 | 79.5 | 21.8 | 0.00382 |
| LDCH [mg/dL] | 104.1 | 30.0 | 79.5 | 21.8 | 0.00382 |
| LDFC [mg/dL] | 31.0 | 7.8 | 24.1 | 6.9 | 0.00414 |
| LDHD [-/-] | 1.9 | 0.8 | 1.5 | 0.6 | 0.05096 |
| LDPL [mg/dL] | 60.9 | 14.2 | 48.9 | 10.9 | 0.00368 |
| LDPN [nmol/L] | 1229.8 | 335.9 | 994.6 | 224.0 | 0.00815 |
| LDTG [mg/dL] | 18.1 | 4.2 | 19.0 | 7.4 | 0.33910 |
| TBPN [nmol/L] | 1464.5 | 393.7 | 1291.5 | 285.0 | 0.06766 |
| TPA1 [mg/dL] | 156.5 | 32.6 | 159.4 | 37.3 | 0.40614 |
| TPA2 [mg/dL] | 36.7 | 5.2 | 37.5 | 6.9 | 0.36281 |
| TPAB [mg/dL] | 80.5 | 21.6 | 71.0 | 15.7 | 0.06763 |
| TPCH [mg/dL] | 201.0 | 33.2 | 179.4 | 28.1 | 0.02204 |
| TPTG [mg/dL] | 99.5 | 36.1 | 147.3 | 143.5 | 0.11581 |
| V1CH [mg/dL] | 5.6 | 2.8 | 9.3 | 10.4 | 0.10123 |
| V1FC [mg/dL] | 1.9 | 1.1 | 3.4 | 3.8 | 0.08162 |
| V1PL [mg/dL] | 4.3 | 2.6 | 8.2 | 9.9 | 0.08009 |
| V1TG [mg/dL] | 22.6 | 15.8 | 48.4 | 69.0 | 0.09012 |
| V2CH [mg/dL] | 3.4 | 1.5 | 4.0 | 3.2 | 0.24082 |
| V2FC [mg/dL] | 1.2 | 0.7 | 1.7 | 1.7 | 0.17493 |
| V2PL [mg/dL] | 3.1 | 1.4 | 4.2 | 3.4 | 0.13086 |
| V2TG [mg/dL] | 10.2 | 5.6 | 15.2 | 14.4 | 0.11342 |
| V3CH [mg/dL] | 3.9 | 2.1 | 4.8 | 3.9 | 0.22088 |
| V3FC [mg/dL] | 1.4 | 0.9 | 2.0 | 2.1 | 0.14727 |
| V3PL [mg/dL] | 3.6 | 1.8 | 4.7 | 3.6 | 0.15611 |
| V3TG [mg/dL] | 9.8 | 5.5 | 13.5 | 12.1 | 0.14463 |
| V4CH [mg/dL] | 5.6 | 2.8 | 5.7 | 3.2 | 0.47689 |
| V4FC [mg/dL] | 2.1 | 1.3 | 2.3 | 1.6 | 0.34492 |
| V4PL [mg/dL] | 4.5 | 2.1 | 5.0 | 2.9 | 0.28801 |
| V4TG [mg/dL] | 7.7 | 4.2 | 9.5 | 6.1 | 0.17515 |
| V5CH [mg/dL] | 1.5 | 0.7 | 1.3 | 0.6 | 0.17131 |
| V5FC [mg/dL] | 0.8 | 0.3 | 0.8 | 0.5 | 0.43243 |
| V5PL [mg/dL] | 1.7 | 0.7 | 1.6 | 0.8 | 0.35714 |
| V5TG [mg/dL] | 2.5 | 0.8 | 2.6 | 0.8 | 0.42327 |
| VLAB [mg/dL] | 7.2 | 3.2 | 9.1 | 6.4 | 0.15517 |
| VLCH [mg/dL] | 19.9 | 8.9 | 24.9 | 19.4 | 0.18861 |
| VLFC [mg/dL] | 8.4 | 3.5 | 10.6 | 7.9 | 0.16462 |
| VLPL [mg/dL] | 18.7 | 7.8 | 24.3 | 17.5 | 0.13603 |
| VLPN [nmol/L] | 130.5 | 57.4 | 164.8 | 115.5 | 0.15521 |
| VLTG [mg/dL] | 58.9 | 29.5 | 94.7 | 99.3 | 0.09967 |

**Figure S1.** Correlation between 2 years mean blood phenylalanine (Phe) level and concurrent Phe level in 7 PKU patients (historical blood Phe levels measured in dried blood by tandem mass spectrometry).
